# Intelligence and executive function are associated with age at insult, time post-insult, and disability following chronic pediatric acquired brain injury

**DOI:** 10.3389/fneur.2023.1192623

**Published:** 2024-01-05

**Authors:** Anne Elisabeth Brandt, Torstein B. Rø, Torun G. Finnanger, Ruth E. Hypher, Espen Lien, Bendik Lund, Cathy Catroppa, Stein Andersson, Kari Risnes, Jan Stubberud

**Affiliations:** ^1^Department of Clinical and Molecular Medicine, Norwegian University of Science and Technology, Trondheim, Norway; ^2^Children’s Clinic, St. Olavs Hospital, Trondheim University Hospital, Trondheim, Norway; ^3^Department of Clinical Neurosciences for Children, Division of Paediatric and Adolescent Medicine, Oslo University Hospital, Oslo, Norway; ^4^Brain and Mind, Clinical Sciences, Murdoch Children’s Research Institute, Melbourne, VIC, Australia; ^5^Department of Psychology, Royal Children’s Hospital, Melbourne, VIC, Australia; ^6^Department of Paediatrics, University of Melbourne, Melbourne, VIC, Australia; ^7^Melbourne School of Psychological Sciences, University of Melbourne, Melbourne, VIC, Australia; ^8^Department of Psychology, University of Oslo, Oslo, Norway; ^9^Department of Research, Lovisenberg Diaconal Hospital, Oslo, Norway

**Keywords:** acquired brain injury, intellectual ability, executive function, long-term outcome, child, adolescent

## Abstract

**Background:**

Pediatric acquired brain injury (pABI) profoundly affects cognitive functions, encompassing IQ and executive functions (EFs). Particularly, young age at insult may lead to persistent and debilitating deficits, affecting daily-life functioning negatively. This study delves into the intricate interplay of age at insult, time post-insult, and their associations with IQ and EFs during chronic (>1 year) pABI. Additionally, we investigate cognitive performance across different levels of global function, recognizing the multifaceted nature of developmental factors influencing outcomes.

**Methods:**

Drawing upon insult data and baseline information analyzing secondary outcomes from a multicenter RCT, including comprehensive medical and neuropsychological assessments of participants aged 10 to 17 years with pABI and parent-reported executive dysfunctions. The study examined associations between age at insult (early, EI; ≤7y vs. late, LI; > 7y) and time post-insult with IQ and EFs (updating, shifting, inhibition, and executive attention). Additionally, utilizing the Pediatric Glasgow Outcome Scale-Extended, we explored cognitive performance across levels of global functioning.

**Results:**

Seventy-six participants, median 8 years at insult and 5 years post-insult, predominantly exhibiting moderate disability (*n* = 38), were included. Notably, participants with LI demonstrated superior IQ, executive attention, and shifting compared to EI, [adjusted mean differences with 95% Confidence Intervals (CIs); 7.9 (1.4, 14.4), 2.48 (0.71, 4.24) and 1.73 (0.03, 3.43), respectively]. Conversely, extended post-insult duration was associated with diminished performances, evident in mean differences with 95% CIs for IQ, updating, shifting, and executive attention compared to 1–2 years post-insult [−11.1 (−20.4, −1.7), −8.4 (−16.7, −0.1), −2.6 (−4.4, −0.7), −2.9 (−4.5, −1.2), −3.8 (−6.4, −1.3), −2.6 (−5.0, −0.3), and −3.2 (−5.7, −0.8)]. Global function exhibited a robust relationship with IQ and EFs.

**Conclusion:**

Early insults and prolonged post-insult durations impose lasting tribulations in chronic pABI. While confirmation through larger studies is needed, these findings carry clinical implications, underscoring the importance of vigilance regarding early insults. Moreover, they dispel the notion that children fully recover from pABI; instead, they advocate equitable rehabilitation offerings for pABI, tailored to address cognitive functions, recognizing their pivotal role in achieving independence and participation in society. Incorporating disability screening in long-term follow-up assessments may prove beneficial.

## 1 Introduction

Acquired brain injury (ABI) is sustained after birth by traumatic brain injury (TBI) or atraumatic brain insults such as tumor, stroke, hypoxia, or infection (1). Common sequelae following pediatric ABI (pABI) are neurological problems (2), fatigue (3) and cognitive, social, emotional, and behavioral deficits, leading to reduced participation, quality of life, and pervasive problems in everyday functioning (4–7). The majority may experience persistent cognitive impairments, typically affecting processing speed, attention, and memory, but also intelligence (IQ) and particularly executive functions (EFs) (8–16).

Generally, IQ and specifically verbal IQ is regarded as more robust to injury, and thus less commonly impaired (17). However, there are reports of negative consequences on IQ after pediatric TBI (8, 18), brain tumor (14), stroke (19) and brain infections (20). Moreover, the consequences may manifest over time (21–23), mainly affecting nonverbal (perceptual–spatial) skills (24–26). Furthermore, EFs are believed to have the most pervasive and debilitating consequences of pABI (27) as these top-down cognitive processes are necessary for goal-directed and self-regulating behavior (28–33). EFs are typically described as three interrelated, separable processes (29–31): (a) *updating* (i.e., holding information in mind and manipulating it), (b) *shifting* (i.e., flexibly switching perspectives, attention or responses), and (c) *inhibition* (i.e., ignore distraction, focus, and suppress or/resist pre-potent responses). Some have proposed extending the model to highlight the importance of attentional control or *executive attention* (33–36). EFs develop from childhood to late adolescence, through differentiation (37), and by dissimilar trajectories (38, 39). Inhibition, shifting, and executive attention are known to develop rapidly during pre-school years (40–42) before slowing down during adolescence (43, 44), or continue developing during middle childhood and adolescence (45). Unlike the others, updating or working memory (46, 47) seems to have a more prolonged development (38, 48). Yet, adult levels of EFs may not be reached until late adolescence or early adulthood (42, 49). Of note, rapid brain maturation is associated with enhanced vulnerability for insult (50, 51). Even though executive dysfunctions are common following pABI, they often go undetected, are misattributed or not properly addressed (52, 53), this may potentially intensify the dysfunctions over time (54).

Injury mechanisms and pathological processes instigated by ABI, differ significantly between adults and children, with more diffuse (55, 56) and persistent impairments in pABI (18, 57). In pediatrics, the effects of ABI may not cease to evolve after the acute period and initial recovery, but instead go on due to alterations in pace and course of brain maturation, connectivity and the acquisition of new skills (18, 58, 59). Thus, skills may not develop as expected, and given the protracted nature of development, deficits may emerge over time and in some cases, the deficits become fully evident years after the insult (60). The discrepancies to healthy peers, therefore, tend to escalate over time as demands increase (5). Consequently, pABI is recognized as a chronic health condition (61), leading to childhood mortality, morbidity, and acquired disability (62, 63). Surprisingly, and despite the lasting consequences, the pediatric population remains remarkably underserved and understudied in regard to necessary cognitive rehabilitation following pABI (64–68). In order to increase the knowledge base, there is a need to develop a deeper understanding of recovery, rehabilitation, and long-term implications for the pABI population. Research should focus on the distinct features of pABI and the developmental aspects.

High variability in pediatric long-term outcomes (69) makes prediction difficult (70, 71) and may hamper remediation attainment. Developmental stage or age at insult influence long-term cognitive outcome (72). However, the relationship has proven to be complex (73) and non-linear (74). Contradictory findings are likely a result of multiple factors influencing long-term outcomes (70). Associations between early insults and poorer outcomes within IQ, attention, memory, and EFs applies across different pABI samples (18, 20, 23, 75–79), which point to key similarities related to age and development. Anderson and colleagues (72) found the worst and most universal cognitive deficits in pediatric TBI (pTBI) before the age of two, and in contrast more favorable outcomes after the age of seven. Preschool years and adolescence may represent sensitive periods (80, 81) associated with increased vulnerability due to rapid maturation of function or underlying neural networks (60, 70, 78). Focal brain lesions sustained in childhood generally show favorable recovery (82). However, more global and diffuse insults frequently seen in preschoolers (83, 84) appear more threatening to the developing brain (18). Although this knowledge has changed the clinical administration of aggressive cancer treatment in the youngest patients (85, 86), the risk of underestimating the consequences of early insults prevails. To highlight periods of critical importance, studies have operated with clinically meaningful categorizations (e.g., preschool, school-aged children, or adolescents). Interestingly, a hypothesis of a clinically meaningful distinction pertaining to insults suffered before and after the age of seven in pTBI showed favorable outcomes in children exceeding 7 years when compared to the younger (18, 25). To our knowledge, this remains to be investigated in a sample of mixed pABI etiologies.

In addition to age, it is important to account for time post-insult given the occurrence during active development and brain maturation (18, 58, 59). In terms of recovery after pABI, the first 2 years post insult have been the most thoroughly studied and there is a general agreement that most cognitive recovery occurs during this time band (5, 8, 87–89). Studies addressing recovery 3 to 4 years post-insult have demonstrated great variability and inconsistent results; plateau of symptoms with persistent deficits (90, 91), stepwise worsening (39), or continued recovery (92). Inconsistent results, particularly related to severity and age at the insult, have also been found in the prolonged recovery phase, excess of 5 years (10, 81, 92, 93), and 10 years post-insult (20, 94, 95).

Several studies have demonstrated poorer cognitive performances with increased severity (8) and a “double hazard” where a more severe insult earlier in development has the most devastating outcomes. This has been demonstrated in TBI (18), tumor (96–98) and brain infections (83). In addition, sex differences may affect outcomes as seen in pTBI (99) stroke (100) and tumor (101). Moreover, there may be environmental factors influencing outcome such as social status (e.g., maternal education) (66) or family unit (i.e., if the child lives in a single versus two-parent households) (102). Moreover, etiology may contribute to dissimilar patterns of dysfunction, which necessitate accounting for potential confounding factors when assessing outcomes (102–104).

Essential to any assessment is the ability to document that symptoms such as cognitive deficits, are associated with functional impairments (105). Persons with ABI struggle with ongoing functional limitations characterized by problems executing activities and with involvement in life situations affecting quality of life and daily functioning (106). There is no consensus on how to measure global functioning, but adult ABI studies, including TBI (107) and atraumatic insults (106), have used the Glasgow Outcome Scale- Extended (GOS-E). Cognitive skills may mediate the effect of ABI on daily functioning (108, 109). Cattelani et al. (110) found that those who were re-employed following TBI, scored 20 points higher on formal IQ tests compared to their non-employed counterparts. However, the relationship between cognitive performance and global functioning is not well understood, warranting studies on cognitive performance at different levels of functional recovery (111).

Problems in daily functioning are often reported following pTBI, and parents report greater adaptive deficits in children with severe insults compared to moderate or mild (112, 113). Importantly, uncovering deficits in everyday functioning is crucial since such deficits in childhood go on and predict delayed or failure to achieve adult milestones (e.g., work, independence, and meaningful relationships) (114, 115). Research employing a single measure of global functioning following pABI is scarce (116) even though there is a developmentally appropriate version of GOS-E, the pediatric version of GOS-E (GOS-E Peds) (117). Despite strong associations to functional independence (measured by Vineland Adaptive Behavior Scale) lending support to its concurrent validity for assessing long-term outcomes (118, 119), GOS-E Peds is rarely used. Prospective pABI studies have demonstrated significant improvement in disability during the first year post-injury, followed by minor subsequent improvement (120, 121). Studies that have employed different measures suggest that GOS-Peds might be more sensitive to change over time (122) and GOS-E Peds is recommended as a global outcome measure (123). In general, good cognition relates to favorable functional outcomes; however, adaptive dysfunction may be present in the context of broadly intact IQ in children (124), and clinical pediatric groups have demonstrated lower adaptive levels than expected from their IQ level (125). Since adaptive behavior heavily relies on planning and organizational skills, investigating executive dysfunctions may help explain everyday functional difficulties (16, 52, 126–130). Neumane and colleagues (122) found that up to 80% of children with severe pTBI had significant disabilities 2 years post-injury. They found that poorer functional outcome was significantly associated with lower IQ and increased EF impairments. This is in keeping with previously demonstrated associations between GOS-E peds and IQ (117) and specific cognitive functions (131).

Collectively, there is an immediate need to enhance the evidence-base following pABI to promote more understanding and assist treatment methods to emerge. As one of few studies, we report on brain injury symptoms, cognitive functioning, and level of global function in the chronic phase (>1 year) of pABI, and a population base including more than two thirds of Norwegian patients assessed for pABI the last decade (132). The data was retrieved from one of the first randomized controlled trials (RCTs) investigating the efficacy of pediatric cognitive rehabilitation. Distinct from most previous studies, the current study aim to investigate hypotheses across different pABI etiologies due to common difficulties in the chronic phase. We aim to disentangle how factors relevant to long-term outcomes, namely age at insult and time post-insult are associated with cognitive outcomes adjusting for factors previously shown to have prognostic value. Moreover, we propose novel insight into cognitive performance at different levels of global functioning in daily life. Based on previous studies, suggesting that preschool age is a particularly sensitive period, we use a clinically relevant categorization investigating if early insult (EI, ≤ 7 years of age) is associated with poorer IQ and EFs when compared to late insult (LI, > 7 years of age). We hypothesize that EI will be associated with poorer IQ and EFs. Moreover, we take into account ongoing developmental and recovery processes, by assessing the association between time-bands post-insult and IQ and EFs, hypothesizing that the best cognitive performances are within the first 2 years of insult indicative of most spontaneous recovery. Finally, we explore cognitive performance at different levels of global functioning in daily life as categorized by GOS-E Peds.

## 2 Materials and methods

### 2.1 Study setting and design

This study presents baseline data collected from January 2017 through April 2019 from a multicenter parallel RCT (132, 133) from a population base including more than two thirds of Norwegian patients assessed for pABI the last decade and conducted at pediatric university hospitals.

### 2.2 Participants

Eligible participants were 10–17 years old, with a verified pABI diagnosis (TBI, brain tumor, stroke, hypoxia/anoxia or brain infections/inflammations), at least 12 months after injury/illness/completed cancer therapy, with reported executive dysfunction in daily life determined by a semi-structured interview developed for this study (133).

Exclusion criteria included: (i) pABI before 2 years of age, (ii) cognitive, sensory, physical, or language impairments impairing the ability to attend regular school (i.e., primarily follow educational goals of peers and regular classroom teaching), thus effectively engage and benefit from the intervention, (iii) pre-insult neurological disease, severe psychiatric disorder and/or use of stimulant medication, (iv) recently detected brain tumor relapse, or (v) not fluent in Norwegian (133). None of the participants had received specific cognitive rehabilitation prior to the study.

### 2.3 Recruitment

Invitation letters were sent to potential participants (*n* = 223) identified by hospital discharge diagnosis and record information from three university hospitals with trauma referral centers for the Central, South-Eastern, and Northern regions of Norway, respectively, covering all children assessed for pABI in 3 out of 4 health care regions in Norway (133). The information letter solicited participants experiencing executive dysfunction in daily life. Following a positive invitation response, written informed consent was obtained from potential participants (>16 years) or primary caregivers. Of the 99 individuals who were considered eligible for a semi-structured interview, 10 participants did not meet inclusion criteria and were excluded, and two participants declined to participate. Once the participants had been randomized (*n* = 87), a baseline assessment of 76 participants was completed. Pre-inclusion attrition included 11 participants (after randomization, before baseline), due to worsening of illness, initiation of drug testing, or intensification of physical rehabilitation (*n* = 9), and new information indicating obvious violation of eligibility not previously communicated (*n* = 2).

### 2.4 Measures

#### 2.4.1 Demographic and injury variables used in analyses

The demographic variables were collected by a systematic interview by a study nurse; *Family unit* is defined as the child’s current living situation as informed by the primary caregiver (i.e., intact family unit = living with both parents). *Maternal education level* serves as a proxy for socioeconomic status and is defined as the highest educational level of the child’s mother. *Etiology* was divided into three categories; brain tumor, TBI and other (i.e., stroke, infection/inflammation, hypoxia). Family unit, maternal education, sex, and etiology may contribute to dissimilar patterns of dysfunction, and thus should be accounted for when assessing outcome (103, 104).

#### 2.4.2 Independent variables

##### 2.4.2.1 Age at insult

Based on expected cerebral maturational spurts increasing vulnerability to insults, the participants are presented according to age at insult: early insult (EI) ≤ 7 years and late insult (LI) > 7 years (25).

##### 2.4.2.2 Time post-insult

The time variable was defined as years after the injury for TBI, years after brain surgery (or if no surgery, years post diagnosis) for brain tumors, and years after hospital admission (for other etiologies). Three clinically meaningful post-insult time categories, expanding on Babikian and Asarnow’s (8) review of outcome after pediatric TBI, presented: 1-2-years, 3-4-years and 5-12-years. The latter time-band represent a less researched period on cognitive and adaptive function (108) also coinciding with the termination of most clinical follow-up programs for pABI in Norway (86).

#### 2.4.3 Outcome measures

*Full scale IQ* was estimated by the subscales Vocabulary, Similarities, Digit Span, Coding, Block Design and Matrix reasoning from the Wechsler Intelligence Scale for Children- Fifth Edition (WISC-V) (134), which is an individually administered test battery (*M* = 100, *SD* = 15, subscales *M* = 10, *SD* = 3).

The *EF* assessments applied are the same tests as used for the RCT (133). Standardized neuropsychological tests were used to assess; *EF updating*, Digit Span (WISC-V), presented as scaled scores (*M* = 10, *SD* = 3) (134), *EF shifting*, Trail Making Test 4 (total time) (TMT4, D-KEFS) presented as scaled scores (*M* = 10, *SD* = 3) (135), *EF inhibition*, the Conners’ Continuous Performance Test, 3ed (CPT-III, Commissions) presented as T-scores (inverted) (*M* = 50, *SD* = 10) (136), and finally *EF executive attention*, the Color Word Interference Test 4 (CWIT4) presented as scaled scores (*M* = 10, *SD* = 3) (135). Applicable to all, higher scores indicate better performance.

All performance-based tests described have demonstrated adequate validity and reliability, and most have been recommended by McCauley et al. (123) as outcome measures for research.

#### 2.4.4 Categorization of disability/recovery

Global function (i.e., ability to resume independent living, education and leisure activities) was assessed with the GOS-E, Pediatric Revision (GOS-E Peds) (117), which is an adaptation of the validated adult version (GOS-E). GOS-E Peds assesses global disability and recovery after brain injury, i.e., functional independence inside and outside the home, capacity for work/school, participation in social and leisure activities, and family and peer interactions (137). Further, it provides developmental specificity in the pediatric population with good predictive, criterion, and discriminative validity (117). It is the recommended global outcome measure in pediatric TBI (123) and more recently, it has been used in studies of other etiologies than pTBI (138). GOS-E Peds consists of 8 levels (from a minimum score of 1 to a maximum score of 8). Level 1 (death) and Level 2 (vegetative state) was not applicable in the current study. Severe disability: Level 3 (lower severe, i.e., always needs support in the home) and Level 4 (upper severe, i.e., sometimes needs support at home or always outside of the home). Moderate disability: Level 5 (lower moderate, i.e., self-contained in school, unable to participate in social activities, or daily intolerable psychosocial difficulties) and Level 6 (Upper moderate, i.e., reduced academic capacity, significant decrease in social/leisure participation, or frequent/weekly psychosocial difficulties). Good recovery: Level 7 (lower good, i.e., slightly reduced social/leisure participation, occasional psychosocial difficulties, or other persisting symptoms), and Level 8 (upper good, i.e., no identifiable difficulties related to the injury). For analysis purposes, we merged lower and upper levels into three levels of disability/recovery (69); severe (level 3–4), moderate (level 5–6) and good recovery (level 7–8).

#### 2.4.5 Test-procedures

Experienced test technicians and psychology students (master level) conducted all assessments. Assessments were limited to 1 day, and the tests were arranged in blocks and the block order were randomly assigned to participants. As such, the order of test administration varied among the participants avoiding a test regime where a particular test was administered the last for all participants. Moreover, participants had frequent breaks and a one-hour lunch break to alleviate tiredness. To compensate for variation associated with multiple assessors, a Standard Operating Procedure (SOP) described the protocol and procedures for assessment, and the test administrators received training from an experienced clinical neuropsychologist.

### 2.5 Ethics statement

Study procedures and monitoring were performed according to ICH Guideline for Good Clinical Practice and Norwegian procedures and regulations for Clinical Trials, described by the Norwegian Clinical Studies Infrastructure Network, https://www.norcrin.no/in-english/. Written informed consent was signed for all participants. The study was approved by the Regional Committees for Medical and Health Research Ethics, Norway (2017/772/REK), and conducted in accordance with principles of the Helsinki Declaration and the standards for Ethical Research Involving Children (ChildWatch International and UNICEF). Clinical Trial Registration No.: NCT03215342.

### 2.6 Statistical analysis

Sample demographics and outcomes are presented descriptively, as numbers and percentages, median and interquartile range (*IQR*), or mean (*M*) and standard deviation (*SD*) as suitable. Separate data are presented for the clinically meaningful division of age at insult (early insult, EI, and late insult, LI) and checked for potential differences between the groups on demographics, insult characteristics, and baseline assessments. Normality assumptions were checked by visual inspection of histograms and residual plots and checked for multi-collinearity and homoscedasticity. No imputation of missing scores was made.

Separate linear regression models were employed for the independent variables (i.e., age and time). We investigated associations between age at insult (EI and LI) and the dependent variables (IQ and EFs) measured by performances in standardized neuropsychological tests. Like age at insult, linear regression modeling was employed to investigate associations between time post-insult (1-2-years, 3-4-years, and 5-12-years) and the dependent variables (IQ and EFs). For each of the analyses we controlled for potential confounders; maternal education, family unit, sex and etiology, in a step-wise inclusion to a multivariable model constituting the adjusted analyses (henceforth referred to as adjusted analyses).

Age at insult and time-post insult may correlate, but collinearity checks did not reveal strong associations. We performed supplementary analyses including both variables in the same multivariable regression model to try to assess their unique contributions to the dependent outcome variables.

IQ and EFs assessed by neuropsychological tests were stratified for different levels of global function (GOS-E Peds categories; severe disability, moderate disability, and good recovery). In addition, regression analyses investigating the association between early vs. late insults and IQ and EFs were stratified by global function (disability level). All test scores presented are age-corrected standardized scores (scaled scores or T-scores) with norms from test providers. All statistical testing employed an alpha of 0.05 (two-tailed). Due to the explorative nature of the study, no adjustments for multiple comparisons were made (139), and *p*-values between 0.01 and 0.05 should be interpreted with caution. IBM-SPSS Statistics version 27 and Stata 16 were used for data analyses.

## 3 Results

### 3.1 Sample characteristics

In total, 76 children and adolescents with a median age of 13 years (*IQR*; 11 to 15) were included. A slight majority of the participants were females (57%) and two-thirds lived in intact family units. Sixty-one percent of the mothers had a university or college degree. The median age at insult was 8 years (*IQR*; 5.5 to 10.5). Thirty-three participants (43%) were categorized with an early insult (EI; ≤ 7 years) and 43 participants (57%) with a late insult (LI; > 7 years). Median post-insult time was 5 years (*IQR*; 3 to 7) in the whole sample, 6 (*IQR*; 5 to 8) in EI, and 3 (*IQR*; 1 to 5) in LI. Brain tumor was the dominant cause of insult, diagnosed in 29 participants (38%). Approximately two-thirds had received critical care at the time of insult, with a median of 2 days at an intensive care unit (*IRQ*; 1 to 7), the proportions of participants that had been admitted to intensive care at the time of insult were evenly distributed between EI and LI. All participants had completed either computed tomography (CT), and/or magnetic resonance imaging (MRI) at some point, and 67 (88%) had abnormal findings. Thirty-five (46% of the entire sample) underwent brain surgery, 25 (86%) in the tumor group, either as the sole procedure or in combination with chemotherapy and/or radiation. Importantly, EI and LI groups were similar with regard to demographics, background, and insult characteristics (Table 1).

**Table 1:** Demographics, injury characteristics and baseline characteristics according to age at insult.

	Early insult (EI) ^a^ *n* = 33	Late insult (LI) ^b^ *n* = 43	Total *n* = 76
*Demographic variables*			
Age at assessment, median *(IQR)*, yrs.	11 (10, 13)	15 (13, 16)	13 (11, 15) ***
Sex, girls, *n (%)*	17 (52)	26 (60)	43 (57)
Intact family unit, *n (%) ^c^*	22 (67)	28 (65)	50 (66)
*Maternal educational level*, *n (%) ^d^*			
Primary or high school	12 (36)	15 (35)	27 (36)
University/ college	19 (58)	27 (63)	46 (61)
*Insult characteristics*			
Age at insult, median *(IQR)*, yrs.	5 (4, 6)	10 (8, 13)	8 (5.5, 10.5)***
Time post-insult, median *(IQR)*, yrs.	6 (5, 8)	3 (1, 5)	5 (3, 7) ***
*Primary injury*, *n (%)*			
Brain tumor	12 (36)	17 (40)	29 (38)
Traumatic brain injury	5 (15)	13 (30)	18 (24)
Other	16 (48)	13 (30)	29 (38)
Stroke	*11 (33)*	*6 (14)*	*17 (22)*
Inflammation	*3 (9)*	*4 (9)*	*7 (9)*
Anoxia	*2 (6)*	*3 (7)*	*5 (7)*
Admitted to intensive care unit, *n (%)*	22 (69)	27 (65)	49 (65)
Admitted intensive care unit, median *(IQR),* days	3 (1, 7)	2 (1, 6)	2 (1, 7)
Confirmatory cerebral imaging, *n (%)* ^e^	31 (94)	36 (84)	67 (88)
RH / LH	11 (33) /13 (39)	17 (40) /15 (35)	28 (37) /28 (37)
Bilateral	8 (24)	9 (21)	17 (22)
Cerebellum	14 (42)	12 (28)	26 (34)
Subcortical white matter	11 (33)	9 (21)	20 (26)
Brainstem	8 (24)	10 (23)	18 (24)
Brain surgery ^f^	16 (48)	19 (44)	35 (46)
Brain surgery in the tumor group	10 (83)	15 (88)	25 (86)
Chemotherapy	4 (12)	7 (16)	11 (14)
Radiation therapy	3 (9)	5 (12)	8 (11)
Received rehabilitation, *n (%)* ^g^	25 (83)	36 (86)	61 (85)
Rehabilitation initiation post insult, median (*IQR*), days	3.5 (1, 7)	4 (2, 7)	4 (2, 7)
*Medical examination and survey at baseline* ^h^			
Disability; Severe/moderate/good recovery, *n (%)*	10 (32) / 13 (42) / 8 (26)	7 (17)/ 25 (60) / 10 (24)	17 (23) / 38 (52) / n (25)
Neurological deficits (yes), *n* (%)	17 (55)	16 (37)	33 (45)
Paralysis, *n (%)*	19 (58)	8 (19)	27 (36) ***
Epilepsy, *n (%)*	5 (15)	0 (0)	5 (7) **
Fatigue, total score, median *(IQR)* ^i^	57 (50, 72)	53 (35, 68)	56 (40, 70)
Clinical fatigue, *n (%)* ^i^	22 (67)	35 (81)	57 (75)
Aid from the Educational Psychological Service (EPS) in school, *n (%)* ^j^	21 (68)	21 (50)	42 (58)
Academic performance, teacher, T score, mean *(SD)* (*n* = 69) ^k^	42 (6.3)	46 (6.5)	44 (6.7) *

IQR, interquartile range; RH, right hemisphere; LH, left hemisphere; SD, standard deviation; EPS, Aid from the Educational Psychological Service, Intergroup differences in significance level: *0.05, **0.01, ***0.001.^a^Sustaining pABI ≤ 7 years: early insult, EI. ^b^ Sustaining pABI > 7 years: late insult, LI. ^c^ Children living with both parents. ^d^ 73 out of 76 mothers (96%) stated their level of education. ^e^ Computed tomography (CT) and/or Magnetic resonance imaging (MRI) was conducted as part of routine clinical treatments. ^f^ In addition to participants with brain tumor, 3 with TBI and 7 with stroke had received brain surgery. ^g^ Rehabilitation defined as any in-patient rehabilitation (e.g., physiotherapy, occupational therapy) post-insult, related to the current diagnosis. ^h^ Medical examination at baseline was conducted as a standard clinical and neurological examination by a physician. ^i^ Fatigue was measured with the Pediatric Quality of Life Inventory-Multidimensional Fatigue Scale (PedsQL MFS, parent report) (140). Parents rated each item according to their child’s function the prior month (e.g., “Feels too tired to spend time with his/her friends”). Presented are reversed total score, linearly transformed to a 0–100 scale, with a clinical cut-off < 70 (141), where higher scores indicate less fatigue. ^j^ Aid from the Educational Psychological Service in the school setting was informed by the primary caregiver. ^k^ Academic performance was provided by the average score of teacher ratings from the Child Behavior Checklist- Teacher’s Report Form (142). The TRF provides scores of children’s performance in academic subjects using a 5-point scale ranging from Ratings: 1 = Far below average, 2 = Below average, 3 = Average, 4 = Above average, and 5 = Far above average. The TRF scores were converted into T-scores, *M* = 50, SD = 10.

Seventy-three participants completed the GOS-E Peds, and out of these 17 (23%) were categorized with severe disability, 38 (52%) with moderate disability, and 18 (25%) with good recovery (Table 1). The proportion categorized with severe disability was twice as high in EI (32%) compared to LI (17%). Moreover, 33 participants (45%) had neurological deficits, 55% in EI, and 37% in LI. Concerning etiology, 17 (61%) in the tumor group had neurological deficits, 4 (24%) with TBI, and 12 (41%) with other etiologies. Twenty-seven participants (36%) reported having paralysis and five participants reported epilepsy, all with epilepsy were in the EI group. Fifty-seven (75%) of the participants obtained scores indicating clinical fatigue (<70), 22 (67%) in EI, and 35 (81%) in LI. Forty-two (58%) participants received aid from the Educational Psychological Service in the school setting, 21 (68%) in EI, and 21 (50%) in LI.

### 3.2 Associations between age at insult and IQ and EFs

**Figure 1:**
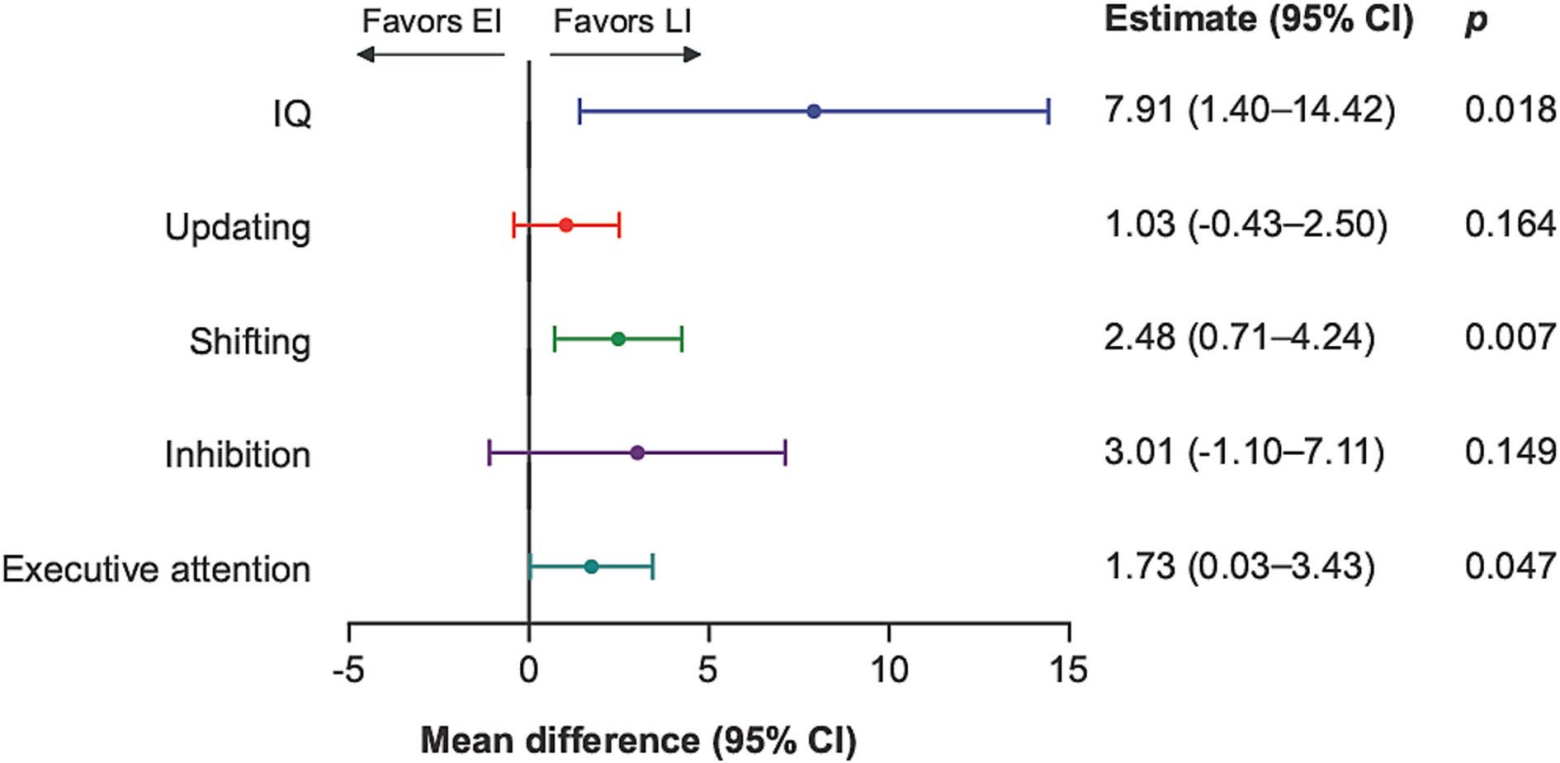
IQ and EFs comparing late insult to early insult with estimated mean difference and 95% confidence intervals. EI, early insult; LI, late insult; CI, Confidence interval. Mean difference of standardized scores, adjusted for demographic variables; maternal education, family unit, sex, and type of insult (etiology) and each endpoint presented according to specified scales.

The mean standardized IQ score among participants was 88 in the EI group and 96 in the LI group. Adjusted for maternal education, family unit, sex, and etiology, the estimated mean difference was 7.9 (95% Confidence Interval, CI: 1.4, 14.4) (Figure 1) (Supplementary Table 1 for the unadjusted analyses).

A total of 23 (32%) participants scored one standard deviation (15 IQ points) or more below the normative mean (100 IQ points), 16 (52%) in EI compared to 7 (17%) in LI. Regarding IQ subtests, the difference between EI and LI was most prominent in the nonverbal tests (Supplementary Table 2).

As with IQ, the adjusted analyses demonstrated better performance with greater age at insult for EFs shifting (mean difference 2.5: 95% CI 0.7, 4.2) and EFs executive attention (mean difference 1.7: 95% CI 0.0, 3.4), but not for updating (mean difference 1.0: 95% CI −0.4, 2.5) and inhibition (mean difference 3.0: 95% CI −1.1, 7.1) (Figure 1).

### 3.3 Associations between time post-insult and IQ and EFs

**Figure 2:**
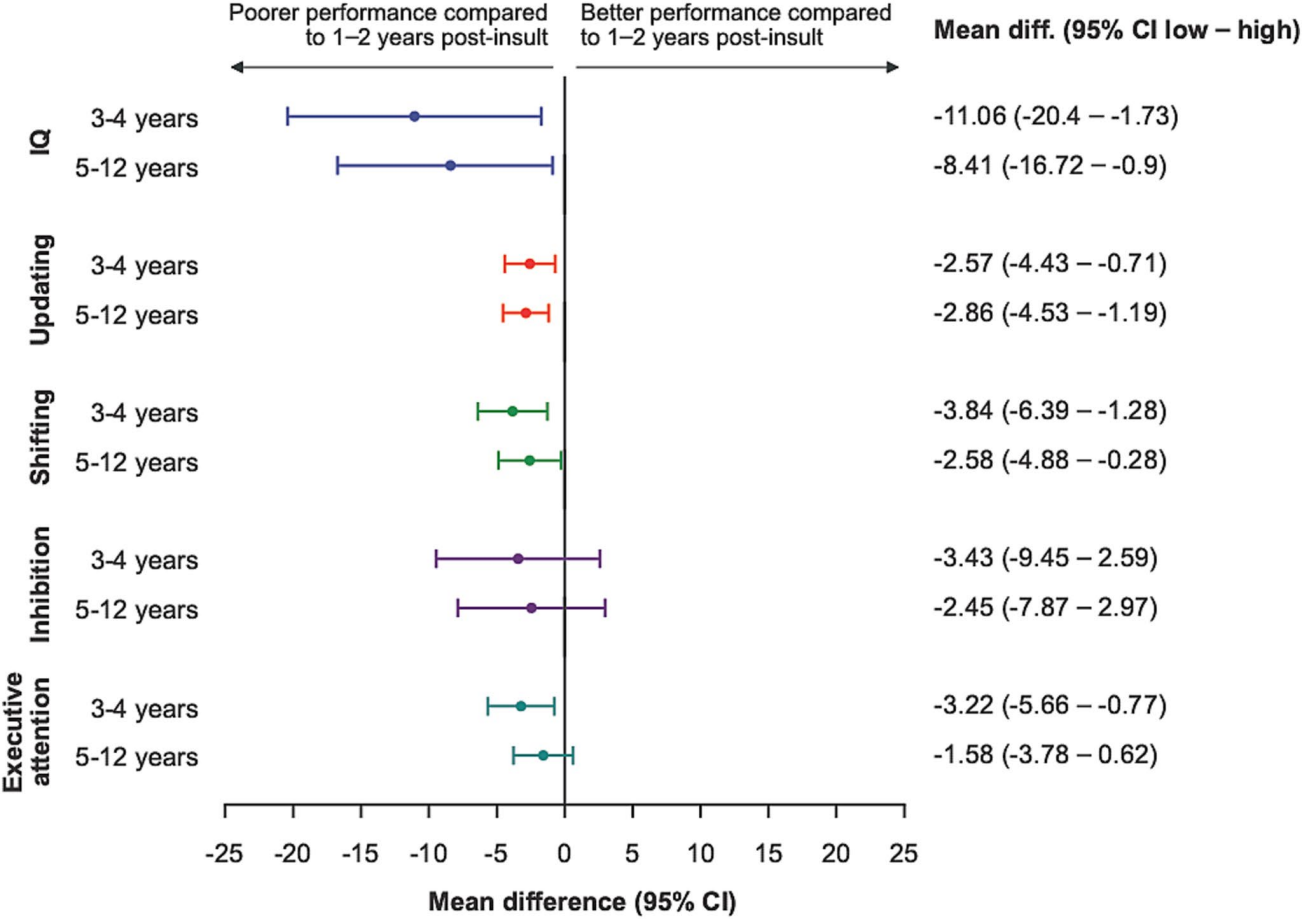
IQ and EFs according to time post-insult when comparing time-bands to 1-2-years post-insult and 95% confidence intervals. CI, Confidence interval. Mean difference of standardized scores, adjusted for demographic variables; maternal education, family unit, sex, and type of insult (etiology) and each endpoint presented according to specified scales.

Compared to 1-2-years post insult, 3-4-years (mean difference −11.1: 95% CI −20.4, −1.7), and 5-12-years (mean difference −8.4: 95% CI −16.7, −0.1) were associated with poorer IQ in adjusted analyses (Figure 2).

Best performance for all EFs was seen in the group with insult within the last 1–2 years. Compared to this group, other time-bands demonstrated poorer performances for EF updating (3–4 –years, mean difference −2.6: 95% CI −4.4, −0.7) and (5-12-years, mean difference −2.9: 95% CI −4.5, −1.2), EF shifting (3-4-years, mean difference −3.8: 95% CI −6.4, −1.3) and (5-12-years, mean difference −2.6: 95% CI −5.0, −0.3) and EF executive attention (3–4 –years, mean difference −3.2, 95% CI −5.7, −0.8) (Figure 2) (Supplementary Table 3 for unadjusted analyses).

Supplementary analyses of mutual adjustments including both age at insult and time post-insult in multivariable regression models demonstrated lower precision, but a largely similar estimate for the age and time effect (Supplementary Table 4).

### 3.4 Cognitive performance at different levels of global functioning in daily life

Participants categorized with good recovery had a mean IQ of 100 (*SD* = 11.62). This was significantly better than moderate disability with a mean IQ of 92.95 (*SD* = 13) and severe disability with a mean IQ of 81.79 (*SD* = 10.62) (Table 2). Controlling for age at insult and time post-insult did not change the levels of significance.

**Table 2:** General intellectual ability and executive functions according to disability level with estimated mean difference and 95% confidence intervals.

	Disability level		
	Good recovery ref. (*n* = 18)	Moderate compared to ref. (*n* = 38) ^a^	Severe compared to ref. (*n* = 17) ^b^
	Mean (*SD*)	Estimated mean difference (95% CI)	Estimated mean difference (95% CI)
IQ	100 (11.62)	−7.05 (−14.1, −0.44)*	−18.2 (−26.9, −9.52)***
IQ ^c^		−7.68 (−14.5, −0.86)*	−17.4 (−25.8, −8.90)***
IQ ^d^		−7.99 (−15.1, −0.87)*	−17.7 (−26.4, −9.07)***
IQ ^e^		−7.64 (−14.7, −0.60)*	−16.7 (−25.3, −8.06)***
*Executive functions*			
Updating, scaled score	10.1 (2.51)	−1.06 (−2.65, 0.54)	−2.72 (−4.67, −0.78)**
Updating, scaled score ^c^		−1.15 (−2.73, 0.44)	−2.64 (−4.57, −0.71)**
Updating, scaled score ^d^		−1.09 (−2.59, 0.41)	−2.30 (−4.09, −0.50)*
Updating, scaled score ^e^		−1.08 (−2.59, 0.44)	−2.26 (−4.08, −0.43)*
Shifting, scaled score	8.7 (2.47)	−0.09 (−2.17, 1.99)	−2.55 (−5.01, −0.09)*
Shifting, scaled score ^c^		−0.27 (−2.31, 1.76)	−2.29 (−4.70, 0.12)
Shifting, scaled score ^d^		−0.55 (−2.61, 1.52)	−2.44 (−4.86, −0.03)*
Shifting, scaled score ^e^		−0.49 (−2.55, 1.56)	−2.17 (−4.60, 0.26)
Inhibition, T-score	49.6 (8.85)	−2.58 (−7.09, 1.93)	−3.61 (−8.94, 1.72)
Inhibition, T-score ^c^		−3.03 (−7.41, 1.36)	−2.99 (−8.18, 2.21)
Inhibition, T-score ^d^		−2.48 (−7.17, 2.21)	−2.90 (−8.38, 2.59)
Inhibition, T-score ^e^		−2.27 (−6.79, 2.26)	−1.81 (−7.17, 3.55)
Executive attention, scaled score	9.6 (2.15)	−1.21 (−2.97, 0.54)	−5.38 (−7.45, −3.31)***
Executive attention, scaled score ^c^		−1.37 (−3.09, 0.35)	−5.16 (−7.20, −3.13)***
Executive attention, scaled score ^d^		−1.49 (−3.28, 0.29)	−5.27 (−7.36, −3.18)***
Executive attention, scaled score ^e^		−1.45 (−3.23, 0.33)	−5.05 (−7.16, −2.94)***

Between group difference; significance level: *0.05, **0.01, ***0.001, ^a^ In moderate disability 37 completed measures of IQ and updating, ^b^ In severe disability 14 completed IQ measures and 15 updating, ^c^ adjusted for age at insult, ^d^ adjusted for time post-insult, and ^e^ adjusted for age at insult and time post-insult.

For all EFs except inhibition, participants with severe disability had a poorer performance compared to those with good recovery (Table 2). In EFs; updating and executive attention, severe disability remained significantly poorer to good recovery, even when adjusting for age at insult, time post-insult or both age and time. For shifting, severe disability remained significantly poorer (compared to good recovery) when adjusting for time post-insult. For inhibition, none of the adjustments resulted in significant differences.

## 4 Discussion

The current study report on brain injury symptoms, neuropsychological functioning, and level of global disability in a heterogeneous chronic pABI sample. The main aim was to examine how developmental factors relevant for long-term outcomes, including age at insult and time post insult, are associated with IQ and EFs. Moreover, we provide novel insight into cognitive performance at different levels of global functioning in daily life using the developmentally appropriate version of GOS-E (GOS-E peds). Our results largely support previous findings with poorer cognitive performance related to early insults (EI: ≤7 years) when compared to later (LI: >7 years) even when adjusting for maternal education, family unit, sex and etiology, thus substantiating this categorization as clinically relevant. Consistent with previous research, our findings demonstrate the best cognitive performances in participants 1–2 years post insult and subsequent poorer performance in those with time post-insult exceeding this. These associations also largely remained significant after controlling for variables such as maternal education, family unit, sex, and etiology. Finally, as one of the first studies, we have examined cognitive performance at different levels of global functioning in daily life. Overall, we found strong associations between cognitive performance and global functioning, with age-expected performances in good recovery and the poorest performance in severe disability level. These associations remained significant after controlling for age at insult and time post injury.

### 4.1 Early insult is associated with poorer IQ and EFs when compared to late insult

#### 4.1.1 General intellectual ability

Our results support an association between EI and poorer IQ as previously shown (8, 14, 18–20, 25, 76, 77, 143, 144). This association withstood adjustment for potential confounding factors such as social status, family unit, sex, and etiology (103, 104, 145). Whereas most past studies have only studied single etiologies, the present study demonstrated associations across different etiologies of pABI. Interestingly, the differences in IQ were primarily due to poorer nonverbal (fluid) skills, as previously shown (24, 25). This is consistent with the assumption of nonverbal IQ as a more sensitive indicator of brain malfunction (146), whereas verbal IQ generally is viewed as more resistant to change and impairment (17).

#### 4.1.2 Executive functions

Our results show that EI is associated with significantly poorer performance on EFs; shifting and executive attention. Since EFs are known to have different developmental trajectories (38, 147) this pattern of results is consistent with the aforementioned EFs having developmental spurts during preschool years (41, 42) and thus, particular vulnerability to insult during such developmental phases (50, 51). Contrary to the other EFs, updating is believed to have a more prolonged developmental trajectory (48) and therefore may be less vulnerable to the influence of early insults. The literature on the effect of age at insult concerning updating (or working memory, WM) is, however, not consistent (148, 149). A meta-analysis suggests that various components of WM may have different degrees of vulnerability to pABI (150). In the current study, we only used a test with resonance to verbal WM (i.e., Digit Span). Gorman et al. (148) investigated both verbal and visual–spatial WM, and even though they found that both modalities of WM were impacted by TBI, younger age at injury was only a significant predictor of poorer performance in visual–spatial WM, thus consistent with our findings. This highlights that there may be modality-specific effects of age at insult concerning updating.

Our results support previous findings that early age at insult serves as a significant risk factor in relation to poorer long-term IQ and most EFs (18, 20, 23, 25, 75–77, 98). Thus, the categorical cut-off (at 7 years) seems to have clinical relevance. Our results are consistent with previous findings of diverse susceptibility to the influence of pABI at different ages (151), believed to reflect the degree of vulnerability in critical stages of neural and cognitive maturation (152). In healthy development, plasticity is considered highly beneficial (i.e., less functional specificity in the immature brain allows transference of functions). However, in the context of brain insults, plasticity may represent a “vulnerability” as predetermined developmental processes are derailed and neural resources are exhausted (153–155). An early insult may diminish cognitive reserve to a greater extent than a later insult, restricting the capacity to support subsequent recovery and development. Insults early in life also influence a less specialized brain and thus have more diffuse and widespread consequences (55, 56) and more persistent impairments (18, 57).

As previously documented, poorer cognitive performances entail long-lasting and global consequences for the acquisition of knowledge, education, and future work as well as independence and function in daily life (13, 109, 126, 129, 130). Based on our findings there is reason to encourage clinicians to be more attentive to children with early pABI and future guidelines should consider age-specific recommendations for follow-up (156).

### 4.2 Time post-insult

#### 4.2.1 General intellectual ability

The highest IQ scores were observed in participants 1–2 years post insult. This finding supports the notion that most recovery occurs during the first 2 years post-insult (87–89), followed by a consistent lag over time in intellectual abilities compared to healthy controls (95). Results indicating poorer IQ at later time points, relative to peers, are consistent with research concerning children following brain tumor and cancer therapy (14, 93, 157). In this group, this decline starts in the first years following the completion of treatment (144). A longitudinal study demonstrated a decline in IQ up to 17.4 IQ points 4 years after ended treatment (98, 158). Another study that included survivors of brain tumor (medulloblastoma) showed that they only attained 49–62% of healthy same-age peers’ achievements (157). The decrease in IQ has been attributed to failure to make age-appropriate gains over time partly due to slower acquisition of knowledge (157), as opposed to actual loss of skills (93). Even though more recent studies have demonstrated that radiotherapy-associated cognitive effects appear to be less attenuated after proton therapies (159), treatment factors in addition to tumor size (160) and tumor pathology (e.g., medulloblastoma) have been associated with lower IQ. Interestingly, a meta-analysis found time since treatment more predictive of IQ than treatment modality (161). In the current study, we did not only demonstrate poorer IQ beyond 1–2 years post insult in those diagnosed with brain tumor, but across pABI etiologies. The results are in agreement with Anderson et al. (95) who demonstrated poorer IQ measured up to 10 years after TBI for both severe and moderate severity compared to healthy controls. These mechanisms are instrumental to the explanation of differential outcomes post-insult.

#### 4.2.2 Executive functions

EF has proven to be one of the last cognitive functions to recover after ABI (52). However, as with IQ, our data point to best performances at 1–2 years post-insult for all EFs, coinciding with the peak in spontaneous recovery (87, 88). Time post-insult exceeding 2 years showed poorer performances, in accordance with previous findings (88, 162, 163). A greater distance to the age norm at later time points may be attributed to increased demands by increasing age (164), perhaps most evident in the school setting. However, the observed distance may depend on the cognitive function being examined and the developmental trajectory of that skill. We found EF-specific patterns, where inhibition seemed less affected by time post-insult with only small differences between time bands compared to the other EFs. As previously noted, updating did not seem as vulnerable to early insults as the other EFs, nonetheless more associated with time post-insult. Our results are in accordance with previous research, indicating inconsistent results concerning the impact of time post-insult on EFs (165). The inconsistency may result from variations concerning different EFs in relation to time post insult and timing of neural development (23). However, larger prospective studies are needed to establish the true significance of time post-insult as difficulties may not be evident until the age at which the skills come “on line” (41).

Finally, the current study has expanded on existing knowledge, exploring associations with IQ and EFs in a mutual adjustment model including both age at insult and time post-insult. These analyses demonstrated lower precision, but a largely similar estimate for the age and time effect. The association between early age at insult and IQ remained relatively unchanged when adjusting for time post-insult. In contrast, the associations between IQ and time post-insult decreased in the same model. This is consistent with TBI studies showing little improvement in performance across time in participants with early injuries (pre-school), compared to older children (18, 166). EFs have shown good predictive power of academic performance in a meta-analysis (167) and the combined impact of lower IQ and impaired learning efficiency may result in poorer academic skills (81). Data from the current study supported poorer teacher-rated performance in EI compared to LI. Further, the association between age at insult and executive attention remained relatively unchanged after controlling for time post insult, while the association between age and inhibition slightly increased after controlling for time. The importance of age is supported by studies that have demonstrated that age at insult predicted performance better than time post-insult (168). However, updating seemed more associated with time post-insult, which the mutual adjustments confirmed. When examining shifting, reductions in all associations was seen when including both age at insult and time post-insult in the model. This may indicate a significant overlap, and an indication of both age and time being relevant for shifting. The association between time and shifting, however, remained statistically significant after controlling for age, and a recent study found evidence for shifting emerging as a more central component in adolescence and adulthood versus childhood (169). It could be that shifting then undertakes a mediational role between inhibition and updating. This information conveys a new insight into the property and the dynamics of EFs, and based on this information it is likely to assume that shifting not only is particularly vulnerable to insult during early years but also during adolescence.

### 4.3 IQ and EFs at different levels of global functioning in daily life

Our findings support a strong association between global functioning in daily life (measured by GOS-E Peds) and IQ (117), with statistically significant poorer IQ in participants both characterized with severe and moderate disability compared to good recovery. The participants categorized with severe disability displayed the clearest evidence of poor EFs, while age-average performance was demonstrated in good recovery. GOS-E Peds has proven sensitive to injury severity in TBI (137) and our results correspond with the results from previous research. Slower recovery and failure to make developmental gains have been demonstrated in severe insults (18, 51, 80, 170, 171). Previous studies on long-term outcome for children with moderate insults have been mixed, demonstrating both decline with increased time post-insult as in severe insults (88) and age-expected performances as in mild insults (94). Even though moderate disability indicates less need for assistance compared to severe, it entails the inability to participate in one or more major areas of activity (i.e., school, leisure, or social activities), thus may have a widespread negative impact (172). Individuals who performed at the higher end on the measures of EF generally tend to require less assistance to be independent (173). However, even in those categorized with good recovery, our results demonstrated large variability in performances. It is possible that subtle changes in adaptive function are not captured that well (174). Additionally, adequate function in daily life despite cognitive impairments may be ensured by the moderation of cognitive reserve (175) or active compensatory strategies (176). Additionally, only looking at group averages from neuropsychological testing may have limitations. Alternative approaches may be the utilization of an impairment index and considering intra-individual performance variability (177).

Previous research has shown an increase in odds for good recovery from 6 to 12 months after pTBI, but not 1 to 5 years post-insult (116). Moreover, functional impairment at 1 year predicted long-term disability up to 7 years post-TBI (122). Both studies suggest a certain stability in functional impairment during the chronic phase (<1-year post-insult) of pABI. Our data do not allow inferences about the stability in disability levels; this should be investigated in future longitudinal studies.

Considering the potential limitations of relying on caregiver report of EF (178), the current study used neuropsychological tests to assess cognitive performance. However, unlike adults, the ongoing developmental processes represent a challenge when investigating cognitive performance in the pediatric population. Although adult intelligence is viewed as a stable trait, intelligence research has profoundly advanced in recent times, pointing to more dynamic mechanisms undergoing extensive developmental changes (179). There are age differences in the development of fluid and crystallized intelligence, with fluid peaking earlier in life than crystallized (146, 180) and young children possess less established skills and knowledge. Further, measuring EFs is associated with several challenges (29, 178, 181, 182). The notion of “task impurity” indicates difficulty when investigating separated EFs, as most tasks require more than one EF process, in addition to non-executive processes (183). Moreover, the EFs described in this study are interdependent and co-occurring (30). Consequently, insults affecting one EF may indeed influence the others as well. Additionally, the ecological validity of performance-based tests has been questioned (41). It has been proposed that tests do not reflect the complexity and demands in real life situations. This can be attributed to the inherently structured and well-defined test situation. Thus, potential dysfunctions may go undetected and discrepancies between test performances and behavior in home and/or school are common (54, 184). The assessment of function in daily living is also difficult. Even though GOS-E Peds has been endorsed for use in clinical research on pABI (117), it measures adaptive abilities and functional outcome in a very broad sense. Unlike the ability to return to work after insult, which has been seen as a success measure in adults, return to school in children does not entail the same significance, as legal mandates require schools to provide education for all children. Tasks that measure actual coping with everyday challenges can be a more valuable tool for a more objective measure of global functioning in further research.

Cognitive functions, and in particular EFs, play a critical role in various daily life activities (185), thus providing sufficient assessments of cognition may be crucial when considering how children actually function in their daily lives after pABI. In fact, cognitive performance may provide a more accurate representation of functional outcome compared to demographic and injury severity variables (186). In adults, cognitive assessments have identified barriers to functional recovery and consequently helped guide cognitive rehabilitation (111, 187). Despite the documented consequences of pABI, many children experience unmet clinical needs (66, 67). As with many previous studies, the current study has shown, that early brain insults have a lasting impact on young lives, and indicate long-term follow-up to detect deficits and provide a contextual understanding of deficits. Moreover, the costs of pABI are greatly affecting patients, families, and healthcare systems (188). Nevertheless, cognitive rehabilitation programs after pABI are still scarce (189–191). This warrant more RCTs in the future aiming at remediating EFs, and providing more equal rehabilitation offers to children and adolescents.

### 4.4 Strengths and limitations

The current study is one of few studies to report on brain injury symptoms, neuropsychological functioning, and level of global disability in a heterogeneous chronic pABI sample. Moreover, we propose novel insight into cognitive performance at different levels of global functioning in daily life using the developmentally appropriate GOS-E peds. The use of standardized testing is considered a strength, and since we utilized baseline data, practice effects were not an issue in this study. Moreover, as all the participants exceeded 12 months post-insult and with a wide range of years post insult, the study presents a broad range of long-term consequences of pABI.

There are also limitations that need to be considered. Since this study analyzed data from a RCT (133), power estimations were not conducted with this study in mind. Further, the cross-sectional design limits inferences of associational directionality and change or developmental trajectories over time. This will be better addressed in longitudinal studies. Additionally, isolating the influence of any one predictor among various confounding and interacting variables represent a known challenge (93). As we have considered both age at insult and time post-insult, which may be confounded to some extent, we have tried to address this analytically by including them in a mutual adjustment model to investigate one while controlling for the other. Further, the study employed categorical quantification of age at insult, time post-insult and disability. Even though the categories in our study build on previous studies and theories reflecting central nervous system growth, they are inevitably inexact and may mask critical developmental periods. Additionally, the categorization of variables may also reduce statistical power (192). The use of population norms as comparisons may also underestimate impairments contrasted to using healthy controls (193). The current study should be viewed as explorative, and our data pointed to consistency in the findings conducting multiple analyses. However, this may inflate the risk of Type 1 errors, thus findings require larger studies to be confirmed. As EFs have shown protracted maturation across development, it is not certain that measures of EFs tap into the same underlying construct across developmental stages (194). Since our study is not prospective, we cannot be certain that the presented data on measurements on EFs tap into the same constructs when individuals are being measured at different ages.

As daily-life executive dysfunction was an inclusion criterion for the RCT, this represents a selection bias. Additionally, a small sample can produce unreliable results. Hence, our results may not be representative of the entire pABI population. Similarly, the study had a slight overweight of female participants, a predominance of non-traumatic injuries, three out of four participants obtained scores indicating clinical fatigue, and the maternal education levels were high, all factors that could influence the representativeness. Interestingly, a relatively small proportion of the participants with brain tumors reported having received chemotherapy and radiation therapy. The eligibility criteria of the RCT which the data was collected from (132, 133) may have contributed to this (i.e., soliciting performance level corresponding to the ability to participate in a metacognitive intervention). This may also indicate a selection bias toward more preserved cognitive function (195–198). On the other side, 65% did receive critical care at an intensive care unit and 88% had pathological imaging indicating more insult severity consistent with the categorization of 75% having moderate or severe disability.

Further, as participants were recruited from the age of 10, those who had experienced an early insult (before the age of 7), had a minimum of 3 years post-insult. Subsequently, this prevented any of the participants with early insults to be in the 1-2-years post-insult group, which may have influenced the results. In addition, insult severity has been established as a well-known predictor of outcome of pTBI (18, 170), but there is no uniform categorization of severity across pABI etiologies. GOS-E Peds have shown sensitivity to injury severity (137), but it is important to bear in mind that it initially was developed to measure outcome after TBI. Accordingly, it may not be as sensitive to non-traumatic insults. As future research would benefit from studying pABI consequences generically (52) there is a need for integration of a uniform categorization of severity in future pABI research. Specifically, brain tumors differ from other pABI etiologies in key areas. Unlike TBI and stroke, tumors often have a more gradual development, more prolonged therapy, the risk of tumor relapse, and the need to restart treatment. Issues as described often exclude children with brain tumor from pABI research (199). The inclusion of various pABI etiologies in the present study may have masked factors specific to one ABI group. Finally, we had a more exploratory approach to the analyses in this study and we have not corrected for multiple comparisons. Therefore, our results must be interpreted with caution and treated as suggestive of possible associations.

## 5 Conclusion

Our findings suggest that early brain insults are associated with poorer performances on IQ and EFs across different pABI etiologies. While confirmation through larger studies is needed, these findings carry clinical implications, underscoring the importance of particular vigilance in diagonstics and rehabilitation of early insults. Moreover, they dispel the notion that children fully recover from pABI; instead, they adcocate for equitable rehabilitation offerings for children and adolescents, tailored to address the cognitive functions most affected, recognizing their pivotal role in achieving independence and participation in society. Finally, we found associations between cognitive performances and level of global functioning, showing age-expected performances in children with good recovery, poorer in moderate and the poorest performance in those with severe disability. Severe disability and in some cases moderate disability, is indicative for rehabilitation needs regarding IQ and most EFs. Disability screening may be a useful tool for identifying those in need of cognitive rehabilitation in the chronic phase of pABI.

## Data availability statement

The raw data supporting the conclusions of this article will be made available by the authors, without undue reservation.

## Ethics statement

The studies involving humans were approved by the Regional Committees for Medical and Health Research Ethics, Norway. The studies were conducted in accordance with the local legislation and institutional requirements. Written informed consent for participation in this study was provided by the participants’ legal guardians/next of kin.

## Author contributions

AB, TR, KR, and JS selected the outcome measures and analyses for the current manuscript. AB conducted the analyses and the initial drafting of the manuscript. AB, TF, and JS conceived the original idea for the RCT. AB, TR, TF, RH, SA, KR, and JS have developed the protocol for the RCT, with contributions from EL, BL, and CC. All authors contributed to the final manuscript and including final approval of the version published.
