# Supplementary material for: Intelligence and executive function are associated with age at insult, time post-insult, and disability following chronic pediatric acquired brain injury
Source: Front Neurol. 2024 Jan 5;14:1192623. doi: 10.3389/fneur.2023.1192623 (PMC10796693; doi:10.3389/fneur.2023.1192623)
Supplement: Supplementary file 1 [file Data_Sheet_1.docx]

|  | total  (*n*=76) | early insult (*n*=33) | late insult (*n*=43) | Estimated mean difference (95% CI) |
| --- | --- | --- | --- | --- |
|  | mean (*SD*) | ref  mean (*SD*) | mean (*SD*) |  |
| IQ (*n*=72) | 92.5 (13.3) | 88.5 (13.25) | 95.5 (12.65) | 7.08 (0.96 to 13.21) * |
| IQ (*n*=72) ^a^ |  |  |  | 7.91 (1.40 to 14.42) * |
| *Executive functions* |  |  |  |  |
| Updating, scaled score | 8.92 (2.84) | 8.35 (2.81) | 9.33 (2.83) | 0.98 (-0.35 to 2.31) |
| Updating, scaled score ^a^ |  |  |  | 1.03 (-0.43 to 2.50) |
| Shifting, scaled score | 8.08 (3.69) | 6.91 (3.79) | 8.98 (3.39) | 2.07 (0.42 to 3.71) * |
| Shifting, scaled score ^a^ |  |  |  | 2.48 (0.71 to 4.24) ** |
| Inhibition, T score | 47.4 (8.25) | 45.24 (7.41) | 49.0 (8.57) | 3.76 (0.03 to 7.49) * |
| Inhibition, T score ^a^ |  |  |  | 3.00 (-1.10 to 7.11) |
| Executive attention,  scaled score | 7.67 (3.58) | 6.52 (3.61) | 8.56 (3.33) | 2.04 (0.45 to 3.63) * |
| Executive attention,  scaled score ^a^ |  |  |  | 1.73 (0.03 to 3.43) * |

**Supplementary**

**Table 1. General intellectual ability and executive functions according to age at insult with estimated mean difference and 95% confidence intervals for unadjusted and adjusted analyses**

*Note*: Between group difference; significance level: *.05, **.01, ***.001, ^a^ adjusted for demographic variables; maternal education, family unit, sex, and etiology.

*CI =* Confidence interval*, SD* = standard deviation

**Table 2. General intellectual ability, including subtests according to age at insult (early insult vs late insult) and estimated mean difference and 95% confidence intervals**

|  | total  (*n*=76) | early insult  (*n*=33) | late insult  (*n*=43) | Estimated mean difference (95% CI) | *P value* |
| --- | --- | --- | --- | --- | --- |
| Cognitive measures,  mean (*SD*) |  |  |  |  |  |
| General intellectual ability  (FSIQ, age normed) (*n*=72) | 92.49 (13.3) | 88.45 (13.25) | 95.54 (12.65) | 7.08 (0.96 to 13.21) | .024 |
| Similarities, scaled score | 9.95 (2.49) | 10 (2.72) | 9.9 (2.32) | -0.1 (-1.27 to 1.08) | .872 |
| Vocabulary, scaled score | 8.64 (2.62) | 8.47 (2.83) | 8.76 (2.48) | 0.29 (-0.94 to 1.53) | .637 |
| Block design, scaled score | 8.49 (3.05) | 7.45 (3.1) | 9.31 (2.77) | 1.85 (0.50 to 3.21) | .008 |
| Matrix, scaled score | 9.44 (2.9) | 8.52 (2.59) | 10.17 (2.95) | 1.65 (0.35 to 2.95) | .013 |
| Digit span, scaled score | 8.92 (2.84) | 8.35 (2.81) | 9.33 (2.83) | 0.98 (-0.35 to 2.31) | .147 |
| Picture span, scaled score | 8.90 (2.97) | 8.68 (3.29) | 9.07 (2.74) | 0.39 (-1.01 to 1.80) | .579 |
| Coding, scaled score | 8.17 (3.19) | 7.61 (2.89) | 8.59 (3.38) | 0.97 (-0.54 to 2.48) | .203 |
| FSIQ ≤ 1 SD (15 IQ points) below norm mean, *n* (*%*) | 23 (32) | 16 (52) | 7 (17) |  |  |

*Note*. *CI =* Confidence interval*, SD* = standard deviation, FSIQ = Full Scale Intelligence Quotient,

|  | Years post-insult | | |
| --- | --- | --- | --- |
|  | 1-2 years  ref  (*n*=17) | 3-4 years  compared to ref  (*n*=19) ^a^ | 5-12 years  compared to ref  (*n*=39) ^b^ |
|  | mean (*SD*) | estimated mean difference (95% CI) | estimated mean difference (95% CI) |
| IQ | 98.71 (12.70) | -10.59 (-19.48 to -1.70)* | -7.27 (-14.87 to 0.32) |
| IQ ^c^ |  | -11.06 (-20.40 to -1.73)* | -8.41 (-16.72 to -0.90)* |
| *Executive functions* |  |  |  |
| Updating, scaled score | 10.71 (2.23) | -2.43 (-4.15 to -0.70)** | -2.49 (-3.98 to -0.99)*** |
| Updating ^c^ |  | -2.57 (-4.43 to -0.71)** | -2.86 (-4.53 to -1.19)*** |
| Shifting, scaled score | 10.24 (2.56) | -3.55 (-5.90 to -1.20)** | -2.34 (-4.38 to -0.29)* |
| Shifting ^c^ |  | -3.84 (-6.39 to -1.28)** | -2.58 (-4.88 to -0.28)* |
| Inhibition, T score | 49.24 (8.33) | -2.71 (-8.23 to 2.81) | -2.47 (-7.27 to 2.34) |
| Inhibition ^c^ |  | -3.43 (-9.45 to 2.59) | -2.45 (-7.87 to 2.97) |
| Executive attention, scaled score | 9.47 (3.62) | -2.94 (-5.26 to -0.63)* | -2.06 (-4.08 to -0.04)* |
| Executive attention ^c^ |  | -3.22 (-5.66 to -0.77)* | -1.58 (-3.78 to 0.62) |

**Table 3. General intellectual ability and executive functions according to time since insult with estimated mean difference and 95% confidence intervals for unadjusted and adjusted analyses**

*Note*. Between group difference; significance level: *.05, **.01, ***.001, ^a^ In the 3-4 years time band 17 completed IQ measures and 18 completed updating, ^b^ In the 5-12 years time band 37 completed IQ and updating measures, ^c^ adjusted for demographic variables; maternal education, family unit, sex, and type of insult (etiology).

*CI =* Confidence interval*, SD* = standard deviation

|  | Models including age *or* time | | | | | | Models including *both* age and time | | | | | |
| --- | --- | --- | --- | --- | --- | --- | --- | --- | --- | --- | --- | --- |
|  | Age at insult  LI compared to EI | | Time post insult  3-4 years compared to 1-2 years | | Time post insult  5-12 years compared to 1-2 years | | Age at insult  LI compared to EI controlling for time | | Time post insult  3-4 years compared to 1-2 years controlling for age | | Time post insult  5-12 years compared to 1-2 years controlling for age | |
|  | *ß* | 95% CI | *ß* | 95% CI | *ß* | 95% CI | *ß* | 95% CI | *ß* | 95% CI | *ß* | 95% CI |
| IQ | 7.9 | 1.4 to 14.4 | -11.1 | -20.4 to -1.7 | -8.4 | -16.7 to -0.1 | 7.0 | -0.5 to 14.6 | -8.2 | -17.8 to 1.5 | -3.8 | -13.4 to 5.7 |
| Updating | 1.0 | -0.4 to 2.5 | -2.6 | -4.4 to -0.70 | -2.9 | -4.5 to -1.2 | 0.3 | -1.3 to 1.8 | -2.5 | -4.4 to -0.5 | -2.7 | -4.7 to -0.7 |
| Shifting | 2.5 | 0.7 to 4.2 | -3.8 | -6.4 to -1.3 | -2.6 | -4.9 to -0.3 | 1.9 | -0.1 to 4.0 | -3.0 | -5.7 to -0.3 | -1.3 | -3.9 to 1.3 |
| Inhibition | 3.0 | -1.1 to 7.1 | -3.4 | -9.5 to 2.6 | -2.4 | -7.9 to 3.0 | 3.3 | -1.6 to 8.1 | -2.0 | -8.4 to 4.3 | -0.3 | -6.6 to 5.9 |
| Executive attention | 1.7 | 0.0 to 3.4 | -3.2 | -5.7 to -0.8 | -1.6 | -3.8 to -0.6 | 1.6 | -0.4 to 3.5 | -2.5 | -5.1 to 0.0 | -0.6 | -3.1 to 2.0 |

**Table 4. Trends in multivariable regression models including both age at insult and time post-insult and adjusted associations with IQ and EFs presented with estimated mean difference and 95% confidence intervals**

*Note*. *CI =* Confidence interval*, SD* = standard deviation
